# The accuracy of the injury severity score in patients below the age of 18 years, a trauma registry data study

**DOI:** 10.1007/s00068-026-03166-9

**Published:** 2026-04-02

**Authors:** Britt H.J. Edwards, Jochem J.P. van Heumen, Stijn D. Nelen, Michael J.R. Edwards, Ruurd L. Jaarsma, Ivo de Blaauw, Erik Hermans

**Affiliations:** 1 Department of Trauma Surgery, Radboud University Medical Centre, Nijmegen, The Netherlands; 2 Department of Orthopedics and Trauma Surgery, Flinders Medical Centre, Adelaide, Australia; 3 Department of Pediatric Surgery, Radboud University Medical Centre, Nijmegen, The Netherlands; 4 Department of Trauma Surgery, Radboud University Medical Centre, Geert Grooteplein Zuid 10, Nijmegen, GA 6525 The Netherlands

**Keywords:** Injury severity score, Trauma score, Pediatric trauma

## Abstract

**Purpose:**

The Injury Severity Score (ISS) is an anatomical score that estimates both severity of trauma and risk of mortality in adults. Although widely used, its applicability in pediatric trauma remains uncertain. This retrospective cohort study evaluated the predictive value of ISS for pediatric mortality and clinically relevant outcomes.

**Methods:**

Patients (< 18 years) treated at a level-1 trauma centre with traumatic injury between 2015 and 2023 were included in the Dutch National Trauma Registry. Receiver operating characteristic (ROC) analyses were used to assess the discriminative performance of ISS for mortality, prehospital advanced care, emergency interventions, imaging, length of stay, surgical burden, and functional outcome.

**Results:**

1733 patients were included. ISS showed excellent discrimination for mortality (AUC 0.97) and good performance for emergency interventions, prehospital advanced care, functional outcome, and admission to higher levels of care, but limited value for predicting number of surgeries. Optimal thresholds for ISS were 16 or higher, except for CT imaging (ISS ≥ 10).

**Conclusion:**

ISS reliably predicts mortality and several clinically relevant outcomes in children. While ISS ≥ 16 remains appropriate for identifying severe injury, optimal thresholds vary by outcome. These findings emphasize the need for outcome-specific interpretation of ISS, for benchmarking and resource allocation.

**Supplementary Information:**

The online version contains supplementary material available at 10.1007/s00068-026-03166-9.

## Introduction

Trauma scores are essential tools used to rapidly triage trauma patients and predict their possible outcomes. A wide range of trauma scoring systems exist, which can broadly be categorized into physiological and anatomical classifications. The accuracy and validity of these scores vary and are influenced by the heterogeneous characteristics of trauma patients, especially in vulnerable groups, such as the pediatric population [1–3].

Anatomical trauma scores, such as the Abbreviated Injury Scale (AIS) and the Injury Severity Score (ISS), focus on injury location and severity, while physiological scores, like the Revised Trauma Score (RTS), evaluate a patient’s immediate prehospital and clinical status. Combined scores, such as the Trauma and Injury Severity Score (TRISS), integrate both anatomical (ISS) and physiological (RTS) data to provide a more comprehensive prognosis in polytrauma patients [1, 2, 4].

Among these, the ISS is one of the most widely utilized anatomical trauma scores. It is calculated by summing the squares of the highest three AIS values, offering a standardized method to assess injury severity across different body regions [5, 6]. The ISS has demonstrated its effectiveness in predicting mortality, morbidity and hospital length of stay in adults. However, its predictive validity for pediatric patients remains uncertain [1, 7, 8].

Pediatric trauma patients present unique challenges compared to adults due to significant differences in anatomy, physiology, and their response to injury. Children’s organs are not fully developed, which can result in divergent outcomes following similar injuries. For instance, a traumatic brain injury may have different consequences for a child than for an adult with the same ISS [9]. Additionally, children’s proportionally larger head size relative to their body mass contributes to an increased vulnerability to high-impact head trauma [10]. Also, initially they may appear hemodynamically stable for an extended period before deteriorating suddenly and rapidly, increasing the difficulty to accurately predict a child’s prognosis. Several traumatic injuries commonly observed in adults, such as rib fractures, occur less frequently in children due to the greater cartilaginous composition of pediatric ribs, which confers increased flexibility. Consequently, children may sustain severe lung contusions without rib fractures, resulting in differing ISS despite comparable underlying thoracic injury [10–13].

One study evaluated mortality prediction using the ISS and TRISS, along with pediatric-specific scores like the Pediatric Risk of Mortality (PRISM). The findings suggest that while PRISM is more sensitive for predicting resource utilization, the ISS may be more reliable for risk-adjusted mortality prediction [14]. Another study showed that a higher 6-hour Glasgow Coma Scale (GCS) score correlates with improved survival rates in pediatric trauma patients, underscoring the potential prognostic value of GCS [15].

Despite the extensive use of trauma scores, there is a limited number of studies assessing their predictive accuracy specifically in pediatric trauma cases. In the Netherlands an ISS ≥ 16 is the conventional category for severe trauma, however, some studies suggest that a higher ISS, such as 25, should be used to classify ‘severe trauma’ in children instead [16]. The ISS provides children with different clinical outcomes than adults for similar ISS values. For example, children demonstrate lower mortality rates than adults for the same ISS. This difference may be partly attributable to the higher prevalence of pre-existing comorbidities in adult populations, which are known to increase the risk of fatal outcomes [17]. Besides increasing the cut-off value for the ISS ‘severe category’, other suggestions to improve the accuracy of this scoring tool have been made. For instance, a weighted ISS system for pediatric blunt trauma patients has a better predictive power for mortality [16, 18–20].

Accurate trauma assessment is critical for both healthcare providers, patients and families. Parents seek reliable prognostic information, while clinicians utilize these scores for benchmarking. Although the ISS is commonly used for pediatric trauma cases, its validity in predicting mortality in children remains unproven. Furthermore, the value in predicting other clinically relevant outcome measures, such as length of hospital stay, hemodynamic stability, and the need for emergency interventions are also unknown.

This study aims to evaluate the effectiveness of the ISS in predicting key outcomes in pediatric trauma patients, including mortality, morbidity, imaging requirements, the necessity for emergency medical interventions, length of hospital stay and intensive care unit stay, and surgery requirement. By assessing the ISS’s predictive capabilities in children, this study seeks to enhance trauma assessment and management strategies for pediatric patients.

## Methods

### Setting and design

**Fig. 1:**
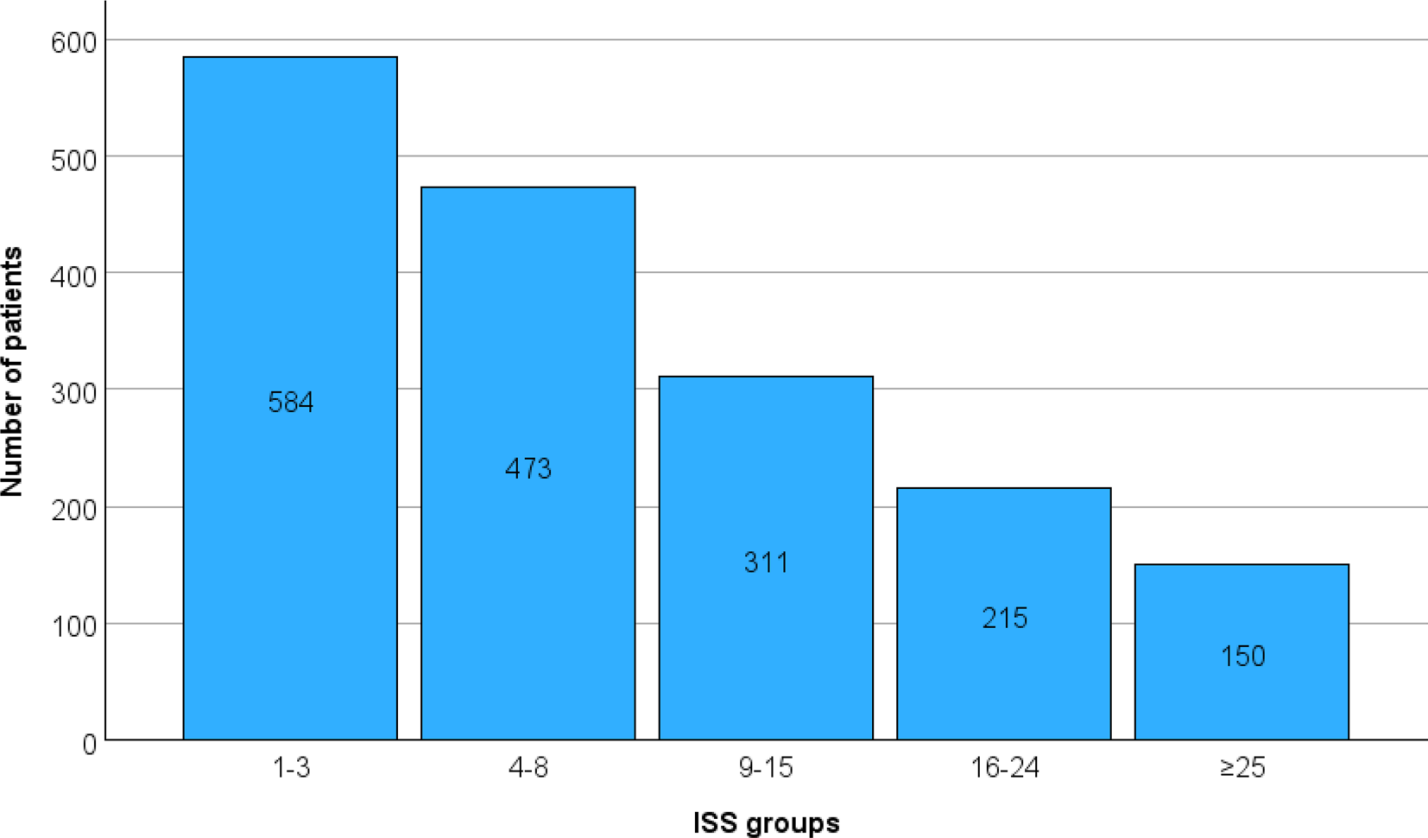
Distribution of participants among the five ISS groups, minor 1–3, mild 4–8, moderate 9–15, severe 16–24 and profound ≥ 25

A single centre, retrospective cohort study was conducted at a Dutch level-I designated pediatric trauma centre including all pediatric patients admitted in hospital between 1st of January 2015 and 1st of January 2023 with any traumatic injury. Various characteristics, prehospital and in-hospital variables were collected in an extensive data registry intended for the Dutch National Trauma Registry database (in Dutch: LTR, Landelijke Trauma Registratie). Patients must be between 0 and 18 years at time of injury and admitted to hospital within 48 h after sustaining their injury. Participants with an unregistered ISS or AIS values, to manually calculate the ISS, were excluded from this study. Patients that were transferred from level-II and level-III hospitals more than 48 h after their injury were also not included in this database. See supplementary information (SI, Fig. 1) for a flow diagram of the inclusion and exclusion criteria.

### Study procedures

The following variables were collected from the LTR database:

- Baseline patient characteristics (age, sex and comorbidities).
- Mechanism of injury.
- ISS and AIS codes.
- Primary recorded vital signs on arrival in hospital, including systolic blood pressure, heart rate, respiratory rate.
- Pre-hospital setting and interventions, such as involvement of MMT (specialized Dutch Mobile Medical Helicopter Team) for on-site intubation or resuscitation (binary variable, requirement of prehospital intervention; yes/no).
- Total length of hospital stay in days and days spent in the intensive care, high care or median care units (IC/HC/MC) (ordinal variable; number of days).
- Glasgow outcome scale at discharge (ordinal variable; good recovery, moderate disability, severe disability, vegetative state and death).
- In hospital mortality (binary variable: yes/no).
- CT scans (binary variable: yes/no).
- Emergency interventions, including damage control thoracotomy, damage control laparotomy, extraperitoneal pelvic packing, extremity revascularization, emergency interventional radiology, craniotomy, ICP monitoring (placement of intracranial pressure monitor), cricothyrotomy, damage control orthopaedic/trauma surgery (e.g. external fixator) and other lifesaving emergency procedures (binary variable, requirement of emergency intervention: yes/no).
- Number of surgeries during admission (ordinal variable; 0, 1, 2, ≥ 3 surgeries).

### Classification of ISS

The ISS was documented by a specialized research data coder of each patient and verified by a recalculation using the AIS values. All patients were stratified into five groups based on their individual ISS ranging from minor to severe trauma. The groups were defined using clinically relevant ISS cutoffs reflecting isolated AIS values, similar to a large cohort study by Bolorunduro et al. [21], to facilitate meaningful interpretation and visualization of the descriptive statistics: ISS 1–3 (minor), ISS 4–8 (mild), ISS 9–15 (moderate), ISS 16–24 (severe), and ISS ≥ 25 (profound). All discriminative analyses were performed using ISS as a continuous variable rather than as categorical subgroups.

### Statistical method

The data was analysed using the IBM SPSS software, version 30.0 for Windows [22] and R software [23], version 4.5.2 for MacOS. Descriptive statistics were used to analyse the patient demographics, using the mean, range and t-tests. After checking for possible confounders in patient characteristics and groups selection, the five ISS subgroups were compared based on all aforementioned variables by performing One-Way ANOVA with post-hoc analysis.

For all binary variables receiver operating characteristic (ROC) curves were made to determine the accuracy of the ISS as a continuous predictor. In addition, a clinically relevant dichotomous cutoff of ISS ≥ 16 was evaluated, reflecting the widely accepted threshold for severe trauma. Sensitivity, specificity at this cutoff were examined to assess its clinical utility and accuracy. Additionally, optimal ISS cutoff values were determined by maximizing Youden’s index on the ROC curve, allowing identification of thresholds that best balanced sensitivity and specificity.

Ordinal variables were evaluated using a cumulative ROC framework. Specifically, a series of binary ROC curves was generated at successive thresholds of the ordered outcome. For example, to assess the ISS predication capability for number of required surgeries, a comparison of patients requiring more than *n* procedures versus those requiring *n* or fewer procedures (*n* = 0, 1, 2 or ≥ 3). For each threshold, the area under the ROC curve (AUC) quantified the probability that a randomly selected patient with a higher ISS had a larger number of surgeries than a randomly selected patient with a lower ISS. Discriminative performance was summarized using both individual AUCs and a macro-averaged AUC.

All AUC values were interpreted as follows: 0.5 indicating no discrimination, 0.7–0.8 moderate discrimination, 0.8–0.9 good discrimination, and values greater than 0.9 excellent discrimination.

### Subgroup analysis

A post hoc sensitivity analysis excluding fatal cases was performed to evaluate the robustness of the association between ISS and length of stay in hospital and IC/MC/HC, given the potential influence of early mortality on length-of-stay estimates. This analysis was performed using cumulative ROC curves. Additionally, to assess potential influence of the injury severity distribution within this cohort on discriminative performance of ISS for mortality, a post hoc sensitivity analyses was performed excluding patients with isolated minor or mild injuries (AIS 1–2, ISS < 9).

## Results

### Participants

1733 patients (1065 boys and 668 girls) with a mean age of 7 years were admitted to a Dutch level-I trauma centre within a timeframe of 8 years, roughly 200 children on a yearly basis (SI, Table 1). The majority of the children (*n* = 584) had a low ISS of 1–3 and the remainder were distributed among the other groups (Fig. 1). There was a significant sex difference among the study population, with males (*n* = 1065) outnumbering females (*n* = 668; χ² = 91.0, *p* < 0.001). However, the proportion of males and females remained consistent across all ISS categories. Despite a gradual increase in mean age with increasing ISS, it remained comparable between groups. Therefore, sex and age were not considered confounding factors in the subsequent analysis of ISS-related outcomes (Table 1).

**Table 1:** Baseline characteristics

Baseline Characteristics	Cases	%
ISS		
1–3	584	33.7
5–8	473	27.3
9–15	311	17.9
16–24	215	12.4
≥25	150	8.7
Sex		
Female	668	38.5
Male	1065	61.5
Age		
0–1 year	360	20.8
2–4 years	328	18.9
5–10 years	465	27.0
11–15 years	374	21.6
16–18 years	206	11.8
Comorbidities (prior to trauma accident)		
Present	196	11.3
Absent	1499	86.5
Unknown	38	2.2
Mechanism of injury		
RTA (Road traffic accident)	473	27.3
Assault with penetrating object	5	0.3
Assault with blunt object	15	0.9
Low impact fall	461	26.6
High impact fall	323	18.6
Explosion	12	0.7
Burns (e.g. chemical, thermal, electrical)	59	3.4
Drowning	31	1.8
Asphyxia	16	0.9
Other	333	19.2
Unknown	5	0.3
Nature of injury		
Penetrating	132	7.6
Blunt	1587	91.6
Unknown	14	0.8

### Injury patterns

Injury patterns varied substantially across pediatric age categories. Head injuries predominated in the youngest age groups, accounting for over half of injuries in children aged 0–1 years and decreasing progressively with age. In contrast, extremity injuries became more common in older children. Thoracic, abdominal, and spinal injuries showed a gradual increase with age, whereas external injuries and other minor trauma were more frequent among infants and toddlers, reflecting age-dependent patterns of pediatric trauma (Table 2). Penetrating injuries within this cohort were limited (132 patients, 7.6%), as shown in Table 1.

**Table 2:**
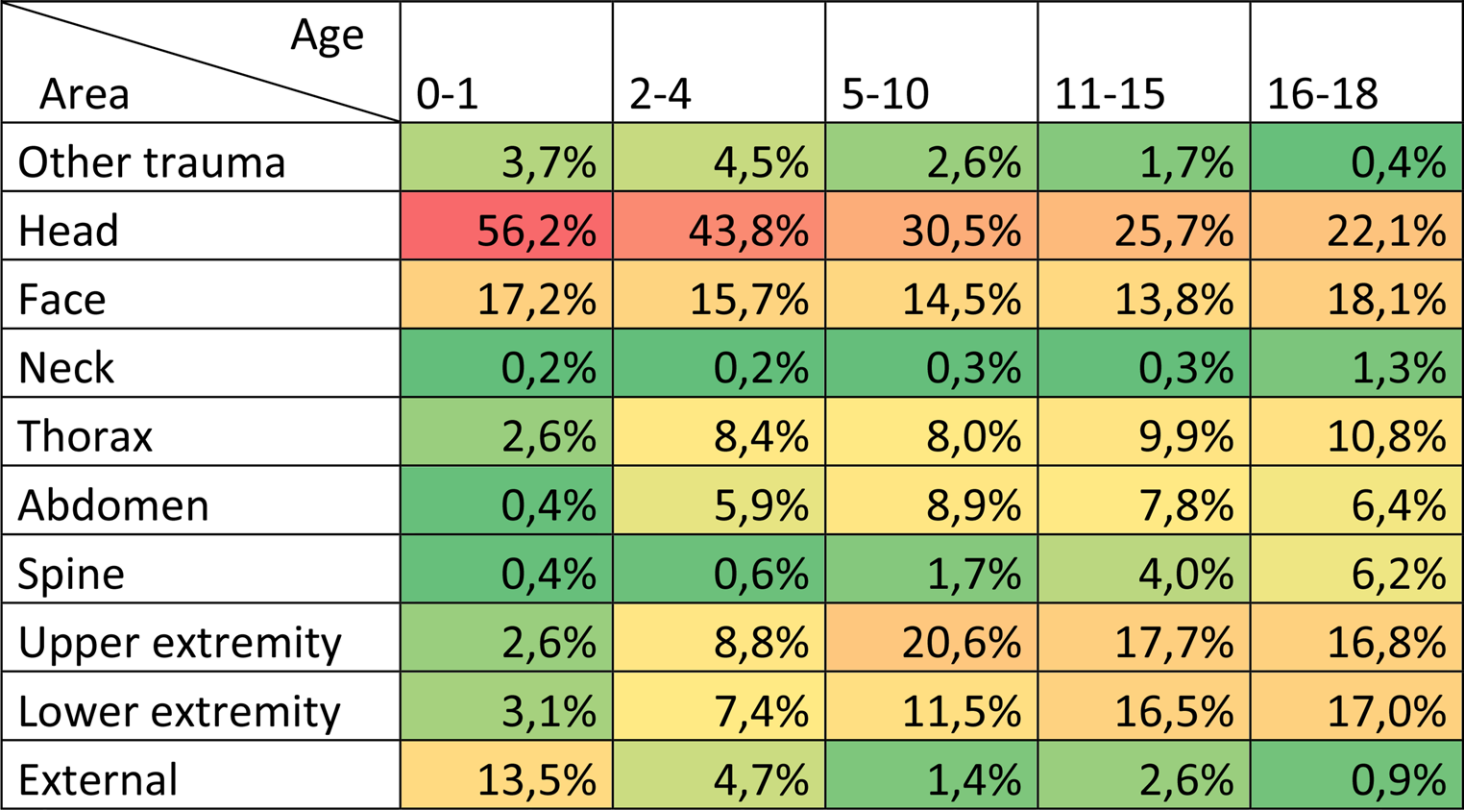
Distribution of injured body regions by trauma type across different pediatric age categories

### Mortality

**Fig. 2:**
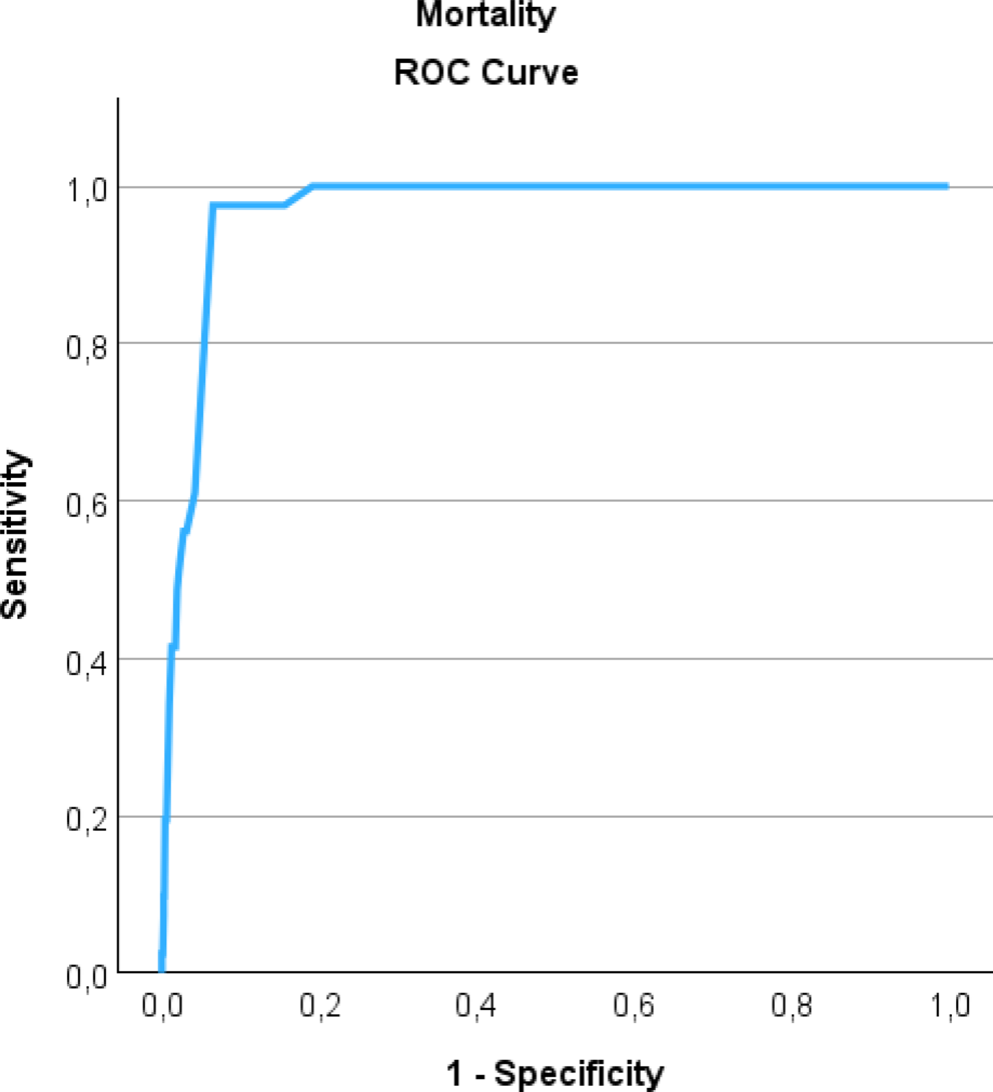
ROC curve showing the validity of ISS predicting risk of in-hospital mortality (AUC 0.97). Sensitivity, specificity at an ISS threshold of ≥ 16 and Youden’s index is displayed in Table 3

Overall, 41 children (2.4%) died during hospital admission, and all fatalities occurred in patients with an ISS ≥ 16. Mortality increased significantly with higher ISS categories. In the ISS ≥ 25 group, 40 of 150 patients (26.7%) died, which was significantly higher than the mortality observed in the ISS 16–24 group (1 of 215 patients, 0.5%). Receiver operating characteristic (ROC) analysis demonstrated excellent discriminative ability of ISS for mortality, with an AUC of 0.97, indicating that higher ISS values are strongly associated with an increased likelihood of in-hospital mortality (Fig. 2). At the clinically relevant threshold of ISS ≥ 16, the sensitivity for mortality was 97.6%, and the specificity was 84.4%, supporting the utility of this cutoff for identifying pediatric patients at high risk of death. However, maximization of Youden’s index for the mortality ROC curve occurred at an ISS ≥ 25, reflecting the threshold with the greatest combined sensitivity and specificity for fatal outcome. The subgroup analyses excluding patients with ISS < 9 (*n* = 1057), the AUC for mortality decreased from 0.97 to 0.92.

### Secondary outcomes

**Fig. 3:**
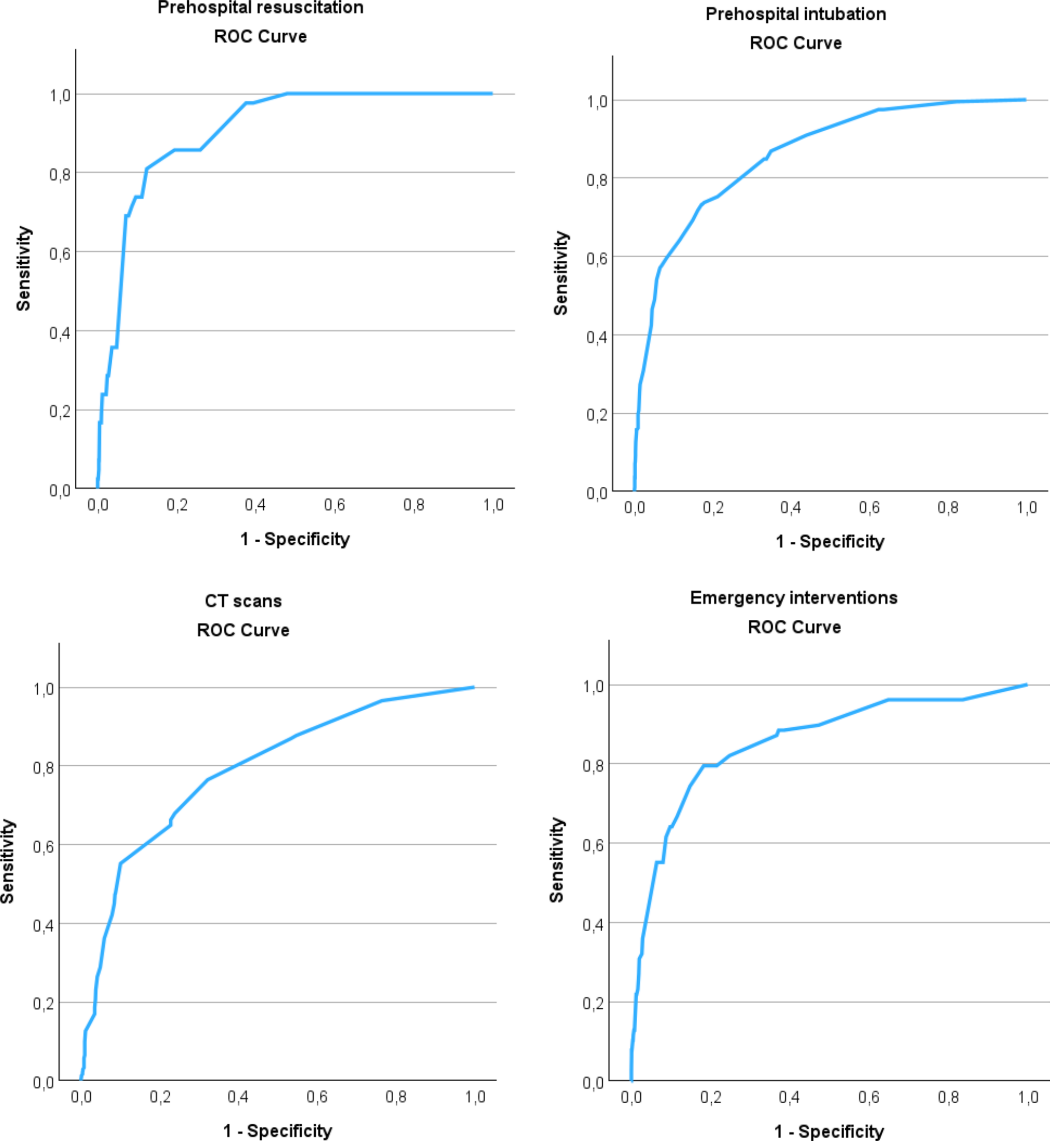
ROC curves showing the validity of ISS predicting all binary secondary outcomes, including prehospital intubation (AUC 0.86), prehospital resuscitation (AUC 0.91), CT scans (AUC 0.79) and emergency interventions (AUC 0.85). Sensitivity, specificity at an ISS threshold of ISS ≥ 16 and Youden’s index is displayed in Table 3

**Fig. 4:**
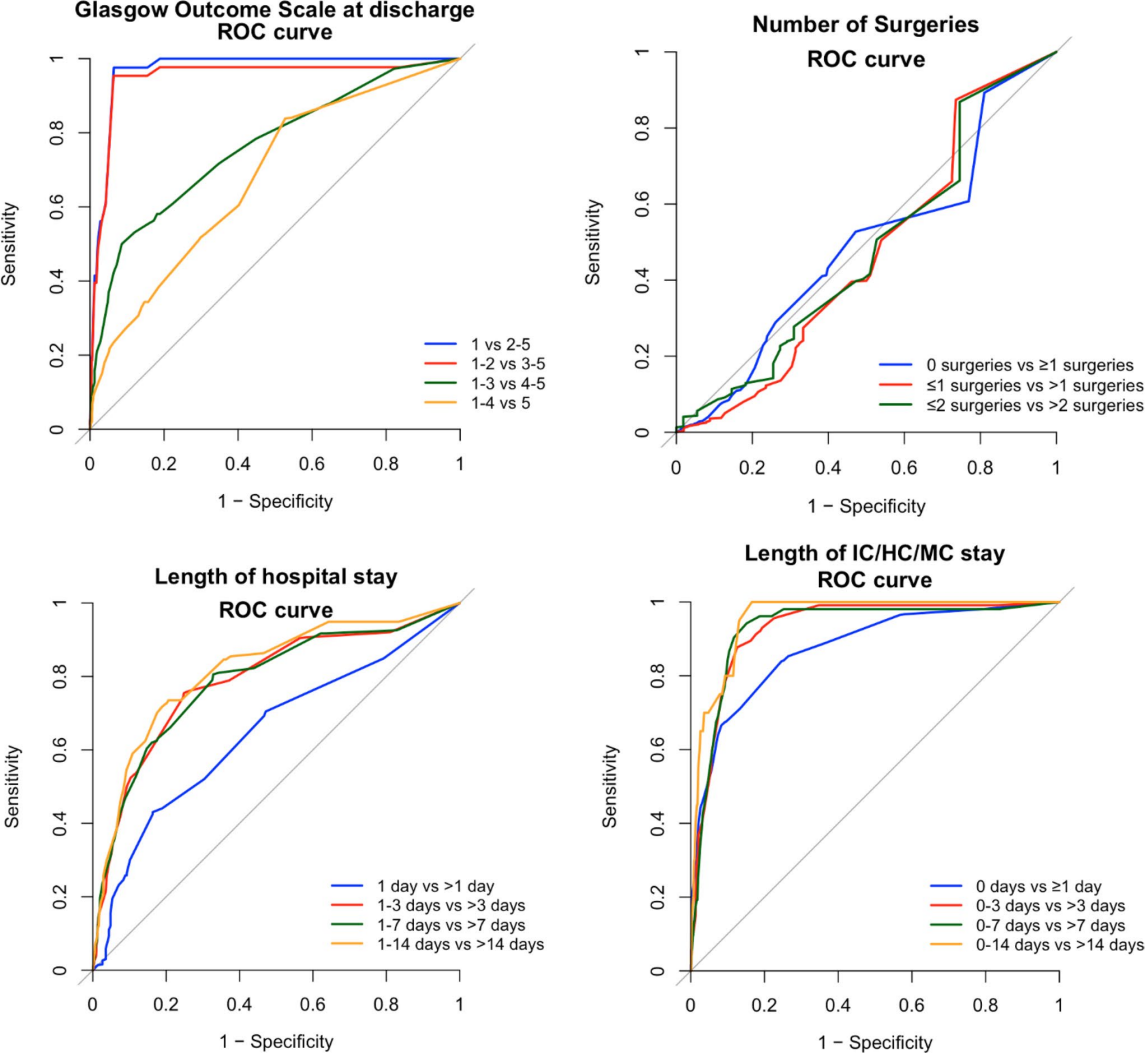
Cumulative ROC curves showing the validity of ISS predicting all ordinal secondary outcomes, including Glasgow Outcome Scale at discharge (macro-averaged AUC 0.84), number of surgeries (macro-averaged AUC 0.48), length of hospital stay (macro-averaged AUC 0.76) and length of stay at intensive care, high care and medium care (macro-averaged AUC 0.92). Corresponding AUC values for each individual curve are shown in Table 4

Binary and cumulative ROC analyses were performed to evaluate the discriminative performance of ISS for all secondary outcomes (Figs. 3 and 4). Additionally, sensitivity and specificity at an ISS threshold of ≥ 16 was calculated and Youden’s index to determine optimal cutoff (Table 3). For ordinal variables the macro-averaged AUC was calculated to interpret the validity of the ISS for these outcomes (Table 4).

### Emergency interventions

In total, 78 patients (4.5%) required emergency interventions in hospital, predominantly consisting of intracranial pressure monitoring, craniotomy and damage control laparotomies. For the prediction of emergency intervention, the ISS demonstrated good discriminatory performance, with an AUC of 0.85. At the ISS threshold of ≥ 16, sensitivity was 74.4% and specificity was 85.1%. The cutoff of ≥ 16 corresponded to the highest Youden’s index, confirming this threshold as the optimal cutoff.

### CT scans

**Fig. 5:**
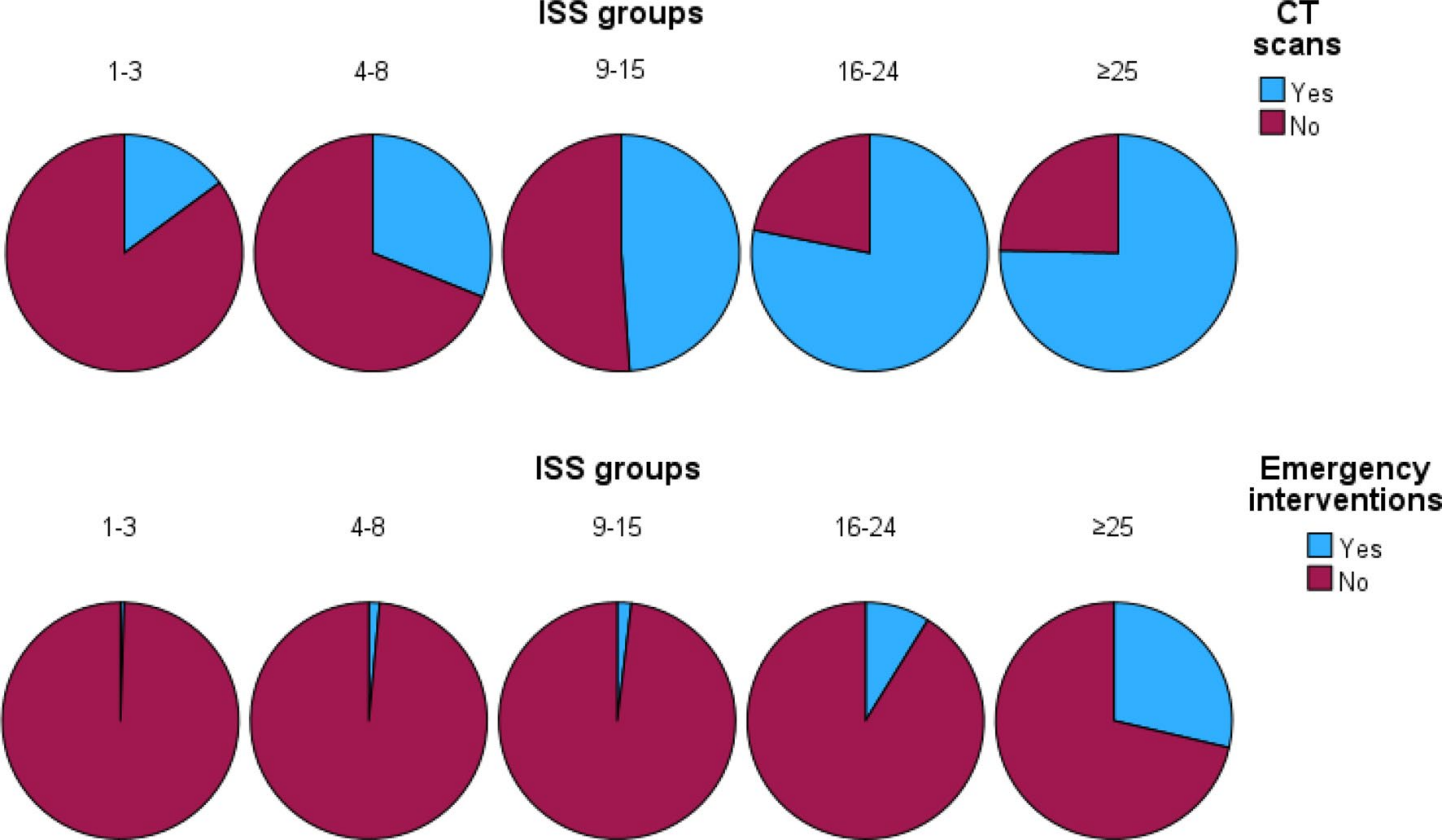
Emergency interventions and CT scans per ISS group. Interventions include damage control surgery/orthopedics, interventional radiology, intercranial pressure measurements, craniotomy and limb (re)vascularization

The predictive performance of ISS for CT scan utilization was moderate with a AUC of 0.79 (Fig. 3). Sensitivity at the conventional ISS ≥ 16 cutoff was very low (36.1%), despite high specificity (94.1%). This discrepancy was further explored using Youden’s index, which identified an optimal ISS threshold of ≥ 10 for CT scan utilization. This divergence in optimal ISS thresholds between CT scan utilization and emergency interventions is illustrated in Fig. 5.

### Prehospital intubation and resuscitation

Prehospital advanced care was frequently required: 198 children (11.4%) were intubated prior to hospital arrival, and 42 patients (2.4%) received prehospital resuscitation, of whom 24 died during hospital admission. The ROC analysis demonstrated that ISS had a good discriminative ability for prehospital intubation, with an AUC of 0.86 and an excellent discriminative ability for prehospital resuscitation, with an ISS of 0.91. This illustrates that requirement of advanced prehospital care is strongly associated to higher ISS values.

### Functional outcome

**Fig. 6:**
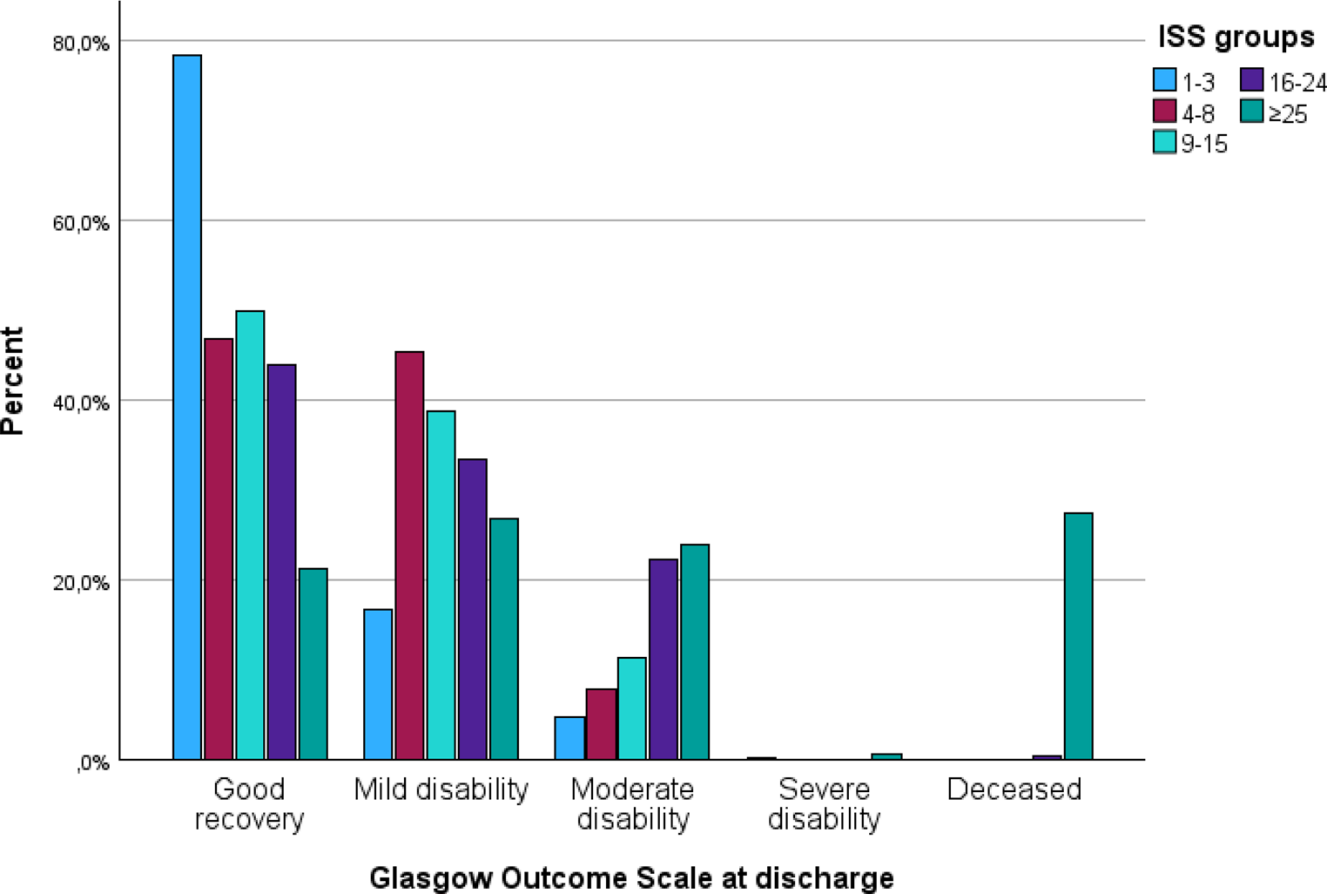
Glasgow outcome scale at discharge per ISS group

In order to provide a global estimate of prognosis after hospitalization following traumatic injury, Glasgow Outcome Scale (GOS) was used to provide insight in the functional outcome upon discharge from hospital (Fig. 6). In this Likert-scale a score of 1 equals death, and 5 shows optimal recovery. ROC analyses showed good discrimination, with an macro-averaged AUC of 0.84, demonstrating a clear association between higher injury severity and poorer neurological and functional recovery.

### Number of surgeries

A total number of 464 children (26.7%) required surgery as part of their treatment plan, of whom 102 patients underwent multiple operations. However, ROC analyses showed no discriminative value of ISS to predict number of surgeries, with an AUC of 0.49.

### Length of hospital and intensive care, high care and medium care stay

Hospital length of stay increased significantly with injury severity. Compared with patients in lower ISS categories, children in the ISS 16–24 group stayed an average of 5 days longer, while those with ISS ≥ 25 remained hospitalized approximately 9 days longer (*p* < 0.001) compared to ISS < 16. The ROC curve showed an AUC value of 0.76, indicating moderate discrimination. Admission to the intensive care, high care and medium care unit occurred significantly more frequently in children with ISS ≥ 16. The macro-averaged AUC was 0.92, indicating good discriminative value of the ISS. Post hoc analysis excluding fatal cases showed a macro-averaged AUC of 0.77 and 0.93 for total hospital stay and IC/HC/MC respectively (Table 4).

**Table 3:** Discriminative performance of ISS, displaying overall area under the curve and sensitivity and specificity at the threshold of ≥ 16 for all binary outcomes

Binary outcomes	AUC	ISS threshold: ≥16		Optimal ISS threshold according to Youden’s index
		Sensitivity	Specificity	
Mortality Emergency intervention CT scans Prehospital intubation Prehospital resuscitation	0.97 0.85 0.79 0.86 0.91	97.6% 74.4% 36.1% 64.1% 83.3%	84.4% 85.1% 94.1% 88.5% 84.1%	0.911 ISS ≥ 25 0.612 ISS ≥ 16 0.451 ISS ≥ 10 0.561 ISS ≥ 13 0.685 ISS ≥ 18

**Table 4:** Discriminative performance of ISS, displaying macro-averaged area under the curve and individual curves for each group for all ordinal outcomes

Ordinal outcomes	AUC
Length of hospital stay	
1 day vs >1 day (blue)	0.65
1-3 days vs ≥3 days (red)	0.79
1-7 days vs ≥7 days (green)	0.79
1-14 days vs ≥14 days (orange)	0.82
Macro-averaged AUC	0.76
Subgroup macro-averaged AUC (excluding fatal cases)	0.77
Length of ICU/HC/MC stay	
0 days vs ≥1 day (blue)	0.88
0-3 days vs ≥3 days (red)	0.93
0-7 days vs ≥7 days (green)	0.93
0-14 days vs ≥14 days (orange)	0.96
Macro-averaged AUC	0.92
Subgroup macro-averaged AUC (excluding fatal cases)	0.93
Number of surgeries	
0 surgeries vs ≥1 surgeries (blue)	0.49
≤1 surgeries vs >1 surgeries (red)	0.47
≤2 surgeries vs >2 surgeries (green)	0.48
Macro-averaged AUC	0.48
Glasgow Outcome Scale at discharge*	
1 vs 2-5 (blue)	0.97
1-2 vs 3-5 (red)	0.95
1-3 vs 4-5 (green)	0.76
1-4 vs 5 (orange)	0.68
Macro-averaged AUC	0.84

*Glasgow Outcome Scale (GOS): 1 = death, 2 = vegetative state, 3 = severe disability, 4 = moderate disability, 5 = good recovery

## Discussion

The findings in this study underscore the strong relationship between the ISS and key clinical outcomes in children. ISS showed an excellent discriminative value for mortality, prehospital resuscitation, length of intensive care, high care and medium care stay. It had good discriminatory capabilities for requirement of emergency interventions, prehospital intubation and Glasgow Outcome Scale at discharge. Furthermore, ISS displayed moderate value as a predictor for length of hospital stay and CT scans. Optimization of sensitivity and specificity using the Youden’s index revealed that the optimal threshold for ISS in this cohort differs depending on each specific variable. These results implicate that ISS remains a useful benchmarking tool in the pediatric population, yet require more nuance in interpretation and outcome-specific consideration.

Several studies have suggested that the current threshold of ISS ≥ 16, that is widely accepted for adult trauma patients, to predict risk of mortality and indicate severe trauma is too low for pediatric patients [16, 24]. For instance, in a large cohort study by Brown, J.B. et al. [16] the authors suggested that the optimal ISS threshold of > 25 for severe injury might be more appropriate to determine risk of mortality. Consistent with these findings, Youden’s index in the present study identified an optimal mortality threshold at ISS ≥ 25, supporting the use of higher ISS cutoffs for mortality prediction. Brown et al. [16] questioned whether mortality represents the most relevant outcome measure in pediatric trauma, given its low overall prevalence, and proposed that resource-based or functional outcomes may be more appropriate. Building on this concept, the present study extends the evaluation by examining a broader range of clinically relevant outcomes and assessing the predictive accuracy of the ISS for each.

ISS demonstrated good predictive performance for several resource-based outcomes, including prehospital advanced care, IC/HC/MC stay, and emergency interventions, that typically require highly specialized resources available at level I trauma centres. These findings suggest that ISS may inform healthcare resource allocation on a population level. To the authors’ knowledge, limited literature exists on the use of ISS to predict resource-based outcomes in pediatric trauma. Notably, optimal ISS thresholds for prehospital intubation and CT imaging were lower than the conventional cutoff of ≥ 16. This may be explained by specific injury mechanisms, such as drowning and asphyxiation, which necessitate advanced airway management despite relatively low ISS values. The low threshold for CT imaging exists in pediatric trauma to avoid missed injuries. Furthermore, ISS showed poor correlation to the number of surgical procedures, indicating that injury severity does not directly translate to operative burden. These findings may be explained by the fact that resource allocation is fundamentally driven by physiological instability, resource availability and differing expert opinions of physicians regarding treatment, rather than anatomic injury pattern. Such practice-related factors may introduce variability and potential information bias that cannot be fully captured within the constraints of a retrospective registry-based study.

In contrast with previous literature, including the large pediatric trauma cohort by Hatchimonji, J.S., et al. [18], ISS only showed moderate discriminatory value for predicting length of hospital stay. This discrepancy can be attributed to two primary factors. First, the study cohort was derived from a single level I trauma centre and did not capture inpatient days following transfer to secondary or local hospitals. Such transfers are common, particularly among polytrauma patients requiring prolonged hospitalization who are often relocated closer to family, thereby leading to an underestimation of total length of stay at higher ISS. Second, patients who died from their injuries were included in the analysis. These patients would likely have required extended hospitalizations had they survived, which may further contribute to an underestimation of overall hospital length of stay, thus weakening the correlation with ISS. The subgroup analysis excluding these fatal cases yielded results comparable to the primary analysis.

Due to the retrospective design of this study, functional outcome assessment was limited to the Glasgow Outcome Scale recorded at hospital discharge. While this measure provides an overall estimate of functional independence, it is largely driven by neurological status and does not account for impairments resulting from injuries to other body regions that may substantially affect quality of life. In contrast, Brown et al. [16] evaluated functional independence using domains such as feeding, locomotion, expression, transfer mobility, and social interaction, offering a more comprehensive assessment. Nevertheless, both their study and the present analysis assessed functional outcomes at the time of hospital discharge. As functional recovery often continues well beyond the acute phase, assessment at discharge may underestimate long-term recovery or persistent disability. Long-term follow-up studies are therefore warranted to determine whether ISS is predictive of sustained functional recovery and health-related quality of life following pediatric trauma.

A key strength of this study lies in the use of a large, standardized national trauma registry, ensuring consistent data collection and high-quality coding of injury characteristics and outcomes. Injury severity was classified using ISS and AIS codes assigned by trained research personnel and verified through recalculation, enhancing the reliability of severity assessment. In addition, the application of robust and appropriate statistical methods, including ROC analyses for both binary and ordinal outcomes with macro-averaged AUCs, allowed for a nuanced evaluation of the discriminative performance of ISS across a broad range of clinical endpoints.

However, the retrospective nature of the study is subject to inherent biases, including incomplete data and selection bias. Only patients admitted within 48 hours of injury were included, and patients transferred from lower-level centres after 48 hours were excluded. Furthermore, although previous studies have reported comparable findings for several outcomes, this investigation was conducted at a single Dutch level I pediatric trauma centre. The cohort included a large proportion of minor ISS cases, which likely attributed to higher AUC values as these patients carry a low baseline risk for adverse outcomes. The reduced AUC for mortality in the subgroup after exclusion of minor injuries highlights that discrimination metrics are influenced by the baseline risk distribution of the selected population. As a result, the generalizability of these findings to other healthcare systems, trauma networks, or non-tertiary care settings may be limited and should be interpreted in the context of underlying population.

The use of the ISS in the acute clinical setting remains a subject of debate, as it is often not immediately available or sufficiently accurate during initial assessment and therefore not suitable for guiding early clinical decision-making. Additionally, it is important to recognize that the relationship between mortality and ISS is not strictly linear. A sum of three lower AIS values may be higher than an isolated high AIS injury, despite potentially higher mortality risk. However, in spite of these limitations, the strong associations observed between ISS and multiple secondary outcomes in this study suggest that ISS may provide valuable prognostic information beyond the primary phase of care. Rather than focusing predominantly on mortality, which is relatively rare in pediatric trauma [16], greater emphasis may be placed on outcomes such as functional recovery, quality of life, length of hospital stay, and the need for rehabilitation services. Given the distinct physiological characteristics and recovery potential of children compared with adults, trauma severity scores such as the ISS should be applied with a pediatric-specific perspective and used to support longer-term clinical planning, resource allocation, and follow-up strategies, rather than solely as predictors of survival. Interpretation and utility of the ISS could be enhanced by combining it with pathophysiological variables, such as age-adjusted vital signs and requirement of prehospital advanced care.

## Conclusion

In this cohort, the ISS demonstrated excellent discriminative ability for mortality and strong predictive performance for several clinically relevant secondary outcomes, including emergency interventions, prehospital advanced care, functional outcome at discharge, and admission to higher levels of care. The conventional ISS threshold of ≥ 16 reliably identified patients at increased risk for mortality and need for urgent intervention, supporting its continued use as a marker of severe injury in pediatric trauma. However, the optimal ISS cutoff varied across outcomes, with lower thresholds providing better discrimination for resource utilization such as CT imaging and higher thresholds for emergency intervention, highlighting that a single ISS cutoff may not be appropriate for all clinical endpoints. Notably, ISS showed limited utility in predicting the number of surgical procedures, underscoring that injury severity does not directly translate to operative burden. Importantly, the ISS should be viewed as complementary to, rather than a substitute for, physiological assessment, as integration of anatomical and physiological data is likely to improve clinical relevance. Overall, these findings suggest that while ISS remains a robust tool for risk stratification, benchmarking and outcome prediction in pediatric trauma, its interpretation should be tailored to specific clinical and resource-based outcomes rather than mortality alone.

## Supplementary Information

Below is the link to the electronic supplementary material.

Supplementary Material 1

## Data Availability

The data used in this study are derived from the Dutch National Trauma Registry (in Dutch, LTR = Landelijke Trauma Registratie) and are not publicly available. However, they may be made available by the corresponding author upon reasonable request after explicit permission from the LTR committee.
