# Supplementary material for: The accuracy of the injury severity score in patients below the age of 18 years, a trauma registry data study
Source: Eur J Trauma Emerg Surg. 2026 Apr 2;52(1):118. doi: 10.1007/s00068-026-03166-9 (PMC13046688; doi:10.1007/s00068-026-03166-9)
Supplement: Supplementary file 1 — Supplementary Material 1 [file 68_2026_3166_MOESM1_ESM.docx]

Supplementary Information (SI)

Supplementary material for ‘Are children small adults according to the ISS? The accuracy of the injury severity score in children. A trauma registry data study.’

**SI Figure 1.** Flow diagram of patient inclusion and exclusion.

| **Database records**  All pediatric trauma patients admitted between 1 Jan 2015 – 1 Jan 2023 at Dutch level-I pediatric trauma centre  (n = 1736) |
| --- |

| **Pre-registration exclusion** • Transfer from level-II or level-III hospital > 48 hours after injury • Admission > 48 hours after injury • Age > 18 years at time of injury |
| --- |

| **Exclusion criteria (n = 3)** • Missing or unregistered ISS/AIS values (preventing ISS calculation) (n=1)  • Duplicates (n=2) |
| --- |

| **Inclusion criteria** • Age 0-18 years at time of injury • Admission ≤ 48 hours after injury • Any traumatic injury |
| --- |

| **Study cohort**  (n = 1733) |
| --- |

**SI Table 1.** Participants included each year in study cohort, divided by gender.

| Year | Females | Males | Total (%) |
| --- | --- | --- | --- |
| 2015  2016  2017  2018  2019  2020  2021  2022 | 85  77  89  103  72  76  83  83 | 148  135  126  121  135  121  137  142 | 233 (13.4%)  212 (12.2%)  215 (12.4%)  224 (12.9%)  207 (11.9%)  197 (11.4%)  220 (12.7%)  225 (13.0%) |
| Total | 668 | 1065 | 1733 |
